# *De novo *sequencing, assembly and analysis of the genome of the laboratory strain *Saccharomyces cerevisiae *CEN.PK113-7D, a model for modern industrial biotechnology

**DOI:** 10.1186/1475-2859-11-36

**Published:** 2012-03-26

**Authors:** Jurgen F Nijkamp, Marcel van den Broek, Erwin Datema, Stefan de Kok, Lizanne Bosman, Marijke A Luttik, Pascale Daran-Lapujade, Wanwipa Vongsangnak, Jens Nielsen, Wilbert HM Heijne, Paul Klaassen, Chris J Paddon, Darren Platt, Peter Kötter, Roeland C van Ham, Marcel JT Reinders, Jack T Pronk, Dick de Ridder, Jean-Marc Daran

**Affiliations:** 1 The Delft Bioinformatics Lab, Department of Intelligent Systems, Delft University of Technology, Mekelweg 4, 2628 CD Delft, The Netherlands; 2 Department of Biotechnology, Delft University of Technology, Julianalaan 67, 2628 BC Delft, The Netherlands; 3 Wageningen University Centre for Biosystems Genomics, Droevendaalsesteeg 1, 6708PB Wageningen, The Netherlands; 4 Plant Research International, Business Unit of Bioscience, cluster Applied Bioinformatics, Droevendaalsesteeg 1, 6708PB Wageningen, The Netherlands; 5 Department of Chemical and Biological Engineering, Chalmers University of Technology, SE-41296 Gothenburg, Sweden; 6 DSM Biotechnology Center, PO Box 1, 2600MA Delft, The Netherlands; 7 Amyris, Inc, 5885 Hollis Street, Suite 100, Emeryville CA 94608, USA; 8 Institute for Molecular Biosciences, Max-von-Laue-Str. 9, Goethe University Frankfurt, D-60438 Frankfurt, Germany; 9 Kluyver Centre for Genomics of Industrial Fermentation, Julianalaan 67, 2628 BC Delft, The Netherlands; 10 Netherlands Bioinformatics Center, 260 NBIC, P.O. Box 9101, 6500 HB Nijmegen, The Netherlands; 11 Platform for Green Synthetic Biology, Julianalaan 67, 2628 BC Delft, The Netherlands; 12 KeyGene N.V, Agro Business Park 90, 6708 PW Wageningen, The Netherlands; 13 Center for Systems Biology, Soochow University, Suzhou 215006, China

## Abstract

*Saccharomyces cerevisiae *CEN.PK 113-7D is widely used for metabolic engineering and systems biology research in industry and academia. We sequenced, assembled, annotated and analyzed its genome. Single-nucleotide variations (SNV), insertions/deletions (indels) and differences in genome organization compared to the reference strain *S. cerevisiae *S288C were analyzed. In addition to a few large deletions and duplications, nearly 3000 indels were identified in the CEN.PK113-7D genome relative to S288C. These differences were overrepresented in genes whose functions are related to transcriptional regulation and chromatin remodelling. Some of these variations were caused by unstable tandem repeats, suggesting an innate evolvability of the corresponding genes. Besides a previously characterized mutation in adenylate cyclase, the CEN.PK113-7D genome sequence revealed a significant enrichment of non-synonymous mutations in genes encoding for components of the cAMP signalling pathway. Some phenotypic characteristics of the CEN.PK113-7D strains were explained by the presence of additional specific metabolic genes relative to S288C. In particular, the presence of the *BIO1 *and *BIO6 *genes correlated with a biotin prototrophy of CEN.PK113-7D. Furthermore, the copy number, chromosomal location and sequences of the *MAL *loci were resolved. The assembled sequence reveals that CEN.PK113-7D has a mosaic genome that combines characteristics of laboratory strains and wild-industrial strains.

## Background

The 1000-dollar genome, an iconic goal in human genomics, is already a reality for the yeast *Saccharomyces cerevisiae *(based on September 2011 quotes from several sequencing companies for sequencing a 12 Mb genome via paired-end short-read sequencing, at over 40-fold coverage).

Although a high quality reference genome of the laboratory strain *S. cerevisiae *S288C has been available since 1996 [1], there are four main reasons to (re)sequence the genomes of other *S. cerevisiae *strains. First, the considerable sequence divergence among *S. cerevisiae *species may cause practical complications, for example, the design of oligonucleotide arrays and cassettes for gene disruption in non-S288C strains. The discovery of > 250,000 polymorphisms in 71 *S. cerevisiae *strains sequenced at low coverage [2] illustrates that this is not a trivial problem. Secondly, although the genomes of *S. cerevisiae *strains appears to be much more strongly conserved than those of other organisms, such as *E. coli *[3], *S. cerevisiae *strains do show physiologically relevant differences in their gene complement. For example, the absence of a functional *MALx3 *gene in *S. cerevisiae *S288C leads to a maltose-negative phenotype, while an atypical *ENA *gene complement renders the laboratory strain CEN.PK113-7D more sensitive to lithium ions [4]. The possible importance of strain-specific genes is illustrated by the identification of a probable horizontal gene transfer event in the *S. cerevisiae *wine strain EC1118, that led to the acquisition of genes from the spoilage yeast *Zygosaccharomyces bailii *[5]. Third, in addition to the presence or absence of coding regions, differences can occur in non-coding regions, such as promoter regions. Knowledge of such differences is essential for the analysis and modelling of regulatory networks in systems biology [6]. Finally, laboratory evolution is rapidly gaining popularity as a tool to analyse genome function and to select for yeast strains with industrially relevant properties [7-11]. Genome comparisons based on mapping short-read data to a distant relative may overlook structural changes. Hence availability of a well-annotated, high-quality reference genome is essential to interpret the changes that occur during laboratory evolution.

Several wild and domestic yeast strains have been sequenced. At the moment, forty-seven genome projects for *S. cerevisiae *have been registered at GenBank from which twenty-eight contain a *de novo *assembled (draft) genome [1,5,12-20].

The isogenic family of CEN.PK strains was developed by crossing of different laboratory strains of *S. cerevisiae *in the 1990's by a consortium of German yeast researchers [21]. A subsequent multi-laboratory study in which four *S. cerevisiae *strains were compared, confirmed that the CEN.PK strains combine good accessibility to classical and molecular genetics techniques with excellent growth characteristics under controlled, industrially relevant conditions [22]. These strains, and in particular the haploid MATa strain CEN.PK113-7D, have since become extremely popular for studies in systems biology [23,24]. Moreover, the excellent growth characteristics of the CEN.PK strains have resulted in their broad application in metabolic and evolutionary engineering studies, for example for the fermentation of pentose sugars [25-28], production of ethanol [29,30] and spirits [31] production of lactate and pyruvate [32,33], production of C_4_-dicarboxylic acids [34], isoprenoids [35,36], and fungal polyketide (6-methylsalicylic acid) [37].

Genomic differences between *S. cerevisiae *CEN.PK113-7D and the S288C strain have been the subject of several studies. Daran-Lapujade and co-workers [38] performed a comparative genotyping of the two strains by hybridization of genomic DNA to oligonucleotide gene-expression arrays. This work led to the identification of several genes that were absent in CEN.PK113-7D, but present in S288C. Schacherer and co-workers [39] employed an oligonucleotide tiling microarray (Affymetrix *S. cerevisiae *Tiling 1.0R array) based on the S288C genome to detect locations of single nucleotide variation (SNV) in order to narrow down the amount of sequencing needed using traditional sequencing approaches and to find genes absent in CEN.PK113-7D such as *RDS1 *and *EHD3*. SNVs in CEN.PK113-7D compared to S288C have previously been characterized by mapping next-generation DNA sequencing data to the S288C reference genome followed by SNV calling [35]. The use of short read (35-bp) sequences and a limited coverage, prohibited detection of insertions and deletions (indels), unique CEN.PK113-7D sequences and structural variations.

The goal of the present study was to make a high-quality assembled and annotated reference genome of *S. cerevisiae *CEN.PK113-7D sequence available to the academic and industrial yeast research communities. Additionally, we aim to compare the CEN.PK113-7D sequence to that of strain S288C and other previously sequenced *S. cerevisiae *strains. To this end, we performed high-coverage sequencing, *de novo *genome assembly, scaffolding and annotation of *S. cerevisiae *CEN.PK113-7D strain. We explored differences with the S288C genome, including single nucleotide variations, small insertions and deletions (indels) and larger structural variation, copy number variation (CNV) and strain-specific sequences and ORFs.

## Results and discussion

### Genome assembly, scaffolding and annotation

The genome assembly of the CEN.PK113-7D strain sequence was performed by combining Illumina (36 M reads, 51 bp, paired-end) and 454 (0.6 M reads, mean length 280 bp) sequencing datasets (see Methods and Additional file 1: Supplementary methods) that together represented more than 150-fold coverage of the genome. A hybrid assembly strategy followed by scaffolding using paired-end read information resulted in 565 scaffolds with a total size of 11.6 Mbp (GenBank BioProject PRJNA52955; http://cenpk.bt.tudelft.nl) (Table 1), which were subsequently placed into chromosomal scaffolds based on homology to S288C. Genes in the CEN.PK113-7D genome were predicted using a combination of *ab initio *and alignment based gene predictors. Combination of predictions by Jigsaw [40] resulted in a total of 5472 ORFs that were predicted with high confidence, comparable to the 5538 genes annotated in S288C [41]. The difference could be attributed to imperfect gene predictions, to missing sequence in the CEN.PK113-7D genome mostly due to repetitive sequences and to genomic content missing from the sequence data due to sequencing bias (e.g. due to nucleotide composition of specific regions). Analysis of the 0.5 Mbp present in S288C but absent in the CEN.PK113-7D draft genome showed that ~26% was in genomic regions with low read mapping probably caused by extreme GC content. Most missing sequence was due to sequence repeats. Out of the 0.5 Mbp absent in the CEN.PK assembly more than ~90% was in repetitive regions. Moreover, about 24 kbp in the CEN.PK assembly was found absent in the S288C genome. This additional sequence will be discussed below.

**Table 1:** Assembly statistics

	454 Newbler contigs	Hybrid Mira contigs	Illumina velvet PE scaffolds	Hybrid velvet PE scaffolds	MAIA integrated PE scaffolds	Chromosomal scaffolds	Contigs not in chromosomal Scaffolds
# contigs/scaffolds > 200	727	2237	647	617	565	16 + 1	55

Largest contig (kbp)	149	284	263	271	353		

N50 (kbp)	39	62	71	73	61		

Total size (Mbp)	11.6	13.0	11.6	11.6	11.6	11.5	0.06

Total size contigs > 200 (Mbp)	11.5	12.8	11.5	11.5	11.6	11.5	0.06

Tiling on S288C	11.01	9.7	10.8	10.6	10.6		

Coverage on S288C	11.5	11.8	11.6	11.6	11.5		

The 454 and Illumina reads were assembled with four approaches (column 1-4). The resulting contig sets were combined into a final assembly with the MAIA algorithm [42]. Ordering and orienting the contigs using homology to the S288C chromosomes resulted in chromosomal scaffolds. The N50 is the length-weighted median of the contig lengths. Coverage on S288C indicates the number of base pairs from the S288C genome covered in the assembly.

### Reassembly and analysis of replicated sequences

A major challenge of *de novo *genome assembly with the current read length of 'next generation sequencing' methods is still the identification and reconstruction of repetitive regions. Regions with sequence similarity within the genome are therefore often left unresolved and cause assembly fragmentation. The approximately 38 Long Terminal Repeat (LTR) transposons in the CEN.PK113-7D genome with a length of about 6 kbp each, as well as the whole genome duplication that occurred in the evolutionary history of *S. cerevisiae *[43] contribute to the presence of repetitive sequences throughout the genome.

*S. cerevisiae *has five retrotransposon families (Ty1-y5) (Additional file 2: Table S1). Each Ty element is flanked by LTR sequences [44]. A total of 50 LTR flanked retrotransposons have been characterized in the S288C strain [1], whereas only 17 retrotransposons were identified in the strain YJM789, a strain isolated from the lung of an AIDS patient with pneumonia [12]. Of the 50 retrotransposons found in S288C [1], evidence of the presence of 40 was obtained in CEN.PK113-7D genome assembly. We found that 39 retrotransposon sequences were indeed located on contig breaks, which suggests an intact transposon or a remnant of one (Additional file 3: Figure S1). A 40th transposon, YCLWTy5-1, was fully *de novo *assembled. YCLWTy5-1 is the only Ty5-type retrotransposon in *S. cerevisiae *(SGD). Analysis of the depth-of-coverage ratio between S288C and CEN.PK113-7D gave a rough estimate of the transposon content and suggested that CEN.PK113-7D had 38 Ty retrotransposons, two fewer than the 40 possible retrotransposons derived from the assembly. This difference might be caused by two contig break locations not containing full-length transposons, possibly due to Ty excision by internal recombination, leaving remnant LTRs.

**Figure 1:**
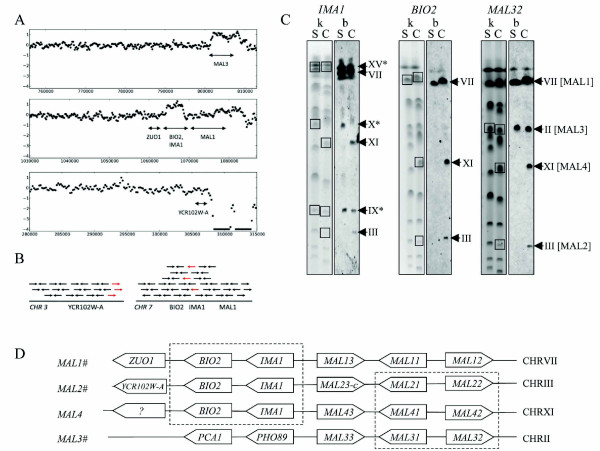
**Overview of the reconstruction of the *MAL2 *locus in *S. cerevisiae *CEN.PK113-7D**. **A**) Depth of coverage analysis of *MAL *loci. Sequencing reads from the CEN.PK113-7D and S288C have been mapped onto the S288C genome sequence. Log_2_-ratio's of the average sequencing depth in 414-bp windows of CEN.PK113-7D over S288C have been plotted versus genomic position. Log_2_-ratio's were capped at -4. **B**) Schematic representation of the paired-end linkage analysis that showed anomalous read pairs (red arrows) on the end of chromosome III that mapped to *BIO2 *on CHRVII. **C**) Southern blots and karyotypes, denoted with 'b' and 'k', respectively, of S288C and CEN.PK113-7D, denoted with 'S' anc 'C', respectively. Probes for *IMA1, BIO2 *and *MAL32 *were amplified with the primers listed in Additional file 5: Table S8. Square boxes indicate hybridized chromosomes. Chromosomes marked with an asterisk (*) depicts cross-hybridization of the IMA1 probe to paralogs of IMA1 (see text). **D**) Schematic organization of the *MAL *loci in CEN.PK113-7D. Loci marked with (#) were individually amplified, sequenced and assembled.

In addition to transposons, paralogs resulting from duplication events form a class of sequences with high similarities that are difficult to assemble *de novo*. To identify duplicated genes, read depths of the CEN.PK113-7D and S288C genomes were compared. Mapping sequencing reads of both S288C and CEN.PK113-7D to the S288C genome allowed the calculation of a depth of coverage ratio at every location on the genome (Figure 1A), which corrects for systematic biases in mapping depth of individual read sets, such as those caused by differences in GC content. This analysis enabled the identification of six replicated regions in the strain CEN.PK113-7D relative to S288C (Table 2). Five groups of genes comprised these regions including i) *BIO2, IMA1*, ii) *RDL1, RDL2*, iii) *MAL33, MAL31, MAL32, YBR298C-A, YBR300C*, iv) *LEU2, NFS1 *and v) *PHO12, IMD2*. To confirm the sequencing results, electrophoretic karyotypes and Southern blots with *BIO2, IMA1, MAL32, RDL1 *and *PHO12 *probes were carried out (Figure 1C and Additional file 4: Figure S2). Each probing experiment confirmed the duplication of the tested genes. The hybridization pattern with the *RDL1 *probe confirmed the presence of an extra copy on chromosome VI (CHRVI) in addition to the expected copy on CHRXV (Additional file 4: Figure S2). The *PHO12 *hybridization pattern in S288C revealed three chromosomes (I, II and VIII) of which only CHRVIII corresponds to *PHO12 *(Additional file 4: Figure S2). Hybridization with chromosomes I and II resulted from cross-hybridization with *PHO11 *(CHRI), *PHO13 *and *PHO5 *(both on CHRII). In addition to the three bands in S288C, CEN.PK113-7D exhibited a fourth hybridization on CHRXI (Additional file 4: Figure S2), which confirms the extra copy found in CEN.PK113-7D sequencing data.

**Table 2:** Copy number variation between the S288C and CEN.PK113-7D genomes was estimated by mapping CEN.PK and S288C reads to the S288C genome using BWA [45]

CNV S288C coordinates						
**CHR**	**start**	**end**	***p*-value**	**Log2 (CEN.PK/S288C)**	**CEN.PK CHR**	**Amplified Genes**

VII	1064291	1068431	1.01 × 10^-100^	0.86	VII, III, XI	*BIO2, IMA1*

XV	849425	850253	1.63 × 10^-21^	0.86	XV, VI	*RDL1, RDL2*

II	805955	808853	3.92 × 10^-76^	0.89	II, III	*MAL32, YBR300C*

II	801609	805541	6.79 × 10^-110^	0.93	II, III	*MAL33, MAL31, MAL32, YBR298C-A*

III	90771	93253	2.15 × 10^-87^	1.07	?	*LEU2, NFS1*

VIII	553415	556313	2.81 × 10^-138^	1.27	VIII, XI	*PHO12, IMD2*

Read depth ratios and *p*-values were calculated using CNV-seq [46]. Regions with a significantly higher read depth in the CEN.PK113-7D genome are reported in this table.

In contrast to S288C, strains from the CEN.PK lineage are able to grow on maltose as sole carbon source. Both the *MAL3 *locus and the *BIO2/IMA1 *locus have double sequence coverage in CEN.PK113-7D (Figure 1A). In S288C the contiguous gene pair *BIO2/IMA1 *is located upstream of the *MAL1 *locus on chromosome VII (Figure 1C). As already observed for *PHO12 *the presence of paralogs complicated the hybridization pattern. *IMA1*, encoding for an isomaltase, has four paralogs in S288C. Therefore, in addition to the hybridization band on CHRVII corresponding to *IMA1*, we observed three more hybridized chromosomes, CHRIX (*IMA3*), CHRX (*IMA4, IMA5*) and CHRXV (*IMA2*). In CEN.PK113-7D, the *BIO2 *and *IMA1 *hybridization patterns consistently revealed not one but two additional copies of the paired genes on CHRXI and CHRIII. In S288C, *BIO2 *and *IMA1 *are physically linked to the locus *MAL1 *(CHRVII). However, CEN.PK paired-end read mapping to the S288C genome established that the *BIO2/IMA1 *duplication was located on the right arm subtelomeric region of chromosome III (Figure 1B). Hybridization chromosomal profiles with the *MAL32 *probes confirmed the co-localization of *BIO2/IMA1 *and *MAL *loci on three chromosomes CHRVII, and CHRIII but also on CHRXI as extra copies of *BIO2 *and *IMA1*. It was therefore hypothesized that the chromosome III *BIO2/IMA1 *genes preceded the *MAL2 *locus as well, given the gene order also observed on the *MAL1 *locus. This hypothesis was validated by long-range PCR amplification of *MAL1, MAL2*, and *MAL3 *in CEN.PK113-7D. The PCR products were sequenced and assembled individually, which was not possible using whole-genome shotgun data because of the high sequence homology. The sequence assembly results confirmed that the additional copy located on chromosome III linked the additional *MAL2 *locus to *BIO2/IMA1*. We were not able to reconstruct the *MAL4 *locus located on CHRXI that seems to share high similarity with CHRIII *MAL2*.

The maltase *MAL22 *and maltose permease *MAL21 *genes are very similar to their *MAL3 *paralogs, but the regulator gene *MAL23 *is very different from both the *MAL1 *and the *MAL3 *locus. This *MAL23 *mutant allele also known as *MAL2-8 C *[47] is responsible for the partial de-repression of the *MAL *genes in CEN.PK strains in the absence of maltose [48]. Interestingly, the two additional *MAL *(2 and 4) loci found in CEN.PK113-7D presented features of the two common loci in S288C (*MAL1 *and *MAL3*). They probably originated from a two-step event with an initial recombination between *MAL1 *and *3 *at *MALx3 *genes, placing *BIO2 *and *IMA1 *in front of *MAL2 *or *4*, followed by a duplication of newly recombined *MAL *locus (Figure 1D).

The last region that was called duplicated contained two genes, *LEU2 *and *NFS1*. However, this duplication could not be confirmed by Southern blotting of karyotype, nor by direct resequencing of the *LEU2 *locus.

### Single nucleotide variation

Single Nucleotide Variation (SNV) and insertions/deletion (indel) analysis was performed by alignment of the CEN.PK113-7D assembly and the reference sequence of S288C. This analysis identified a total of 21,889 SNVs (Additional file 6: Table S2), of which 13,235 were located in 1,843 open reading frames. 4,677 SNVs (35%) resulted in amino acid changes and affected a total number of 1,406 proteins.

An earlier study by Otero et al (2010), restricting their analysis to genes involved in metabolism, revealed that galactose uptake and ergosterol biosynthesis pathways were enriched for non-synonymous SNVs. In addition to confirming these results, our sequencing data enabled the identification of new variations (Additional file 7: Table S4).

**Figure 2:**
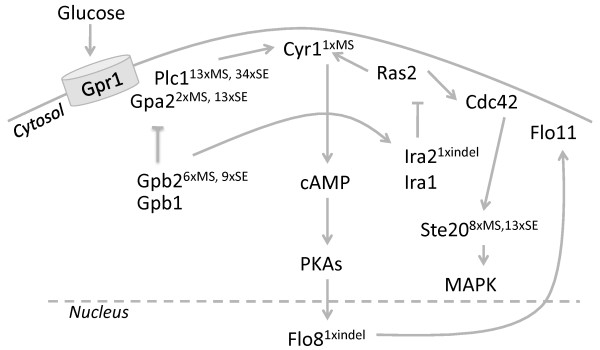
**cAMP signalling pathway in *S. cerevisiae***. Superscript text indicated number of missense (MS), sense (SE) and indel mutations in the genes.

An emblematic mutation in the CEN.PK strain family is located in the *CYR1 *gene, which encodes for adenylate cyclase, a key enzyme for cAMP production and cAMP-dependent protein kinase signalling. The CEN.PK113-7D *CYR1 *gene carries a non-synonymous mutation that results in an amino acid substitution (Lys1876Met) [49]. The metabolic repercussions of this mutation are very limited. It was accompanied by the absence of a cAMP peak and a reduced trehalase activation after sudden exposure to high glucose concentration and a delayed mobilization of storage carbohydrates. While we confirmed the occurrence of this SNV in *CYR1*, several other mutations were found in genes encoding for components of cAMP signalling pathway including *PLC1, GPA2, GPB2, IRA2 *(Figure 2, Additional file 8: Table S5). The *IRA2 *gene exhibits a very early frameshift that disrupts the coding sequence and most importantly the C-terminus of the protein that is essential for its activity [50]. The repercussion of these mutations might be suppressed by the presence of the *lcr1 *(*cyr1*^met1876^) mutation, especially for genes encoding for components of the cAMP signalling pathway located upstream to *CYR1 *(i.e. *GPA2 *and *IRA2*) [49].

In total (including the mutations found in the PKA pathway) 20 genes encoding for proteins involved in mitogen-activated protein kinase (MAPK) signalling pathway (Additional file 9: Figure S4) showed non-synonymous differences with the S288C sequence. The *STE20 *gene implicated in pheromone response, pseudohyphal/invasive growth, vacuole inheritance and down-regulation of sterol uptake carried eight such differences. The gene *MSN2*, involved in the regulation of over 200 genes in response to several stresses, carried one non-synonymous difference [51].

### Small insertions and deletions

In total 2,859 small indels (< 90 bp) were called with an average length of 3 bp. Indels often reside within low-complexity regions, mostly in tandem repeats [52]. 420 indels were found inside a gene, together affecting 297 genes. In 132 genes with indels the start and stop codons usage was not affected (Additional file 10: Table S3).

**Figure 3:**
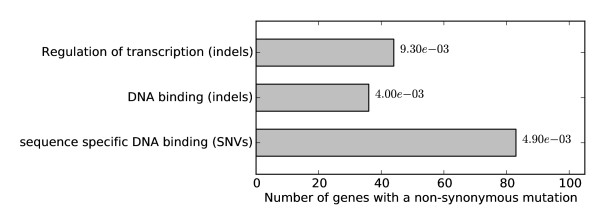
**Enrichment analysis of non-synonymous SNVs (*n *= 1406) and indels (*n *= 297) performed on the Gene Ontology Fat subset and their Bonferroni corrected *p*-values are shown**. All significantly enriched GO terms are shown (α = 0.05).

**Figure 4:**
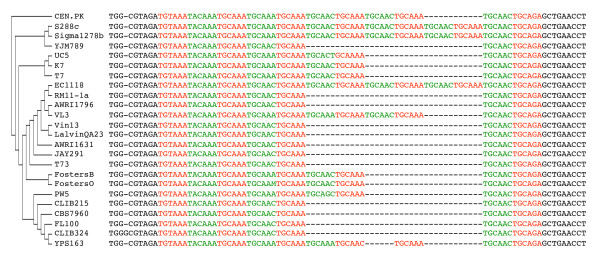
**Tandem repeat variation in *SNF11 *amongst sequenced *S. cerevisiae *strains**. Multiple sequence alignment of *SNF11 *was performed with ClustalW [56]. The cladogram was created using whole genome distances and the UPGMA algorithm. Edge lengths in the cladogram are meaningless. Genbank accessions are listed in Additional file 12: Table S7.

The DAVID functional annotation tool [53] was applied on the genes containing indels to find enrichment of Gene Ontology (GO) terms using the GO Fat subset [54] (Figure 3). The biological process term 'regulation of transcription' (44 genes of 297 genes with indels) was significantly overrepresented (*p *= 9.3 × 10^-3^, Bonferroni corrected). The percentage of gene products involved in 'regulation of transcription' even increased to nearly a quarter of all genes with indels (44 of 220, 20%) when genes classified as uncharacterized or dubious in the *Saccharomyces *Genome Database (SGD) [55] were not considered. Eventually, in 72% (32 of the 44) of the cases, the indel did not cause a frameshift in the transcription factor genes (i.e. start and stop codon usage was conserved). For example, the CEN.PK113-7D *SWI1 *allele carries an in-frame 69 bp deletion, conversely CEN.PK113-7D *SNF11 *contains a 12 bp extension relative to the S288C *SNF11 *allele. Swi1 and Snf11 are both subunits of the Swi/Snf ATP-dependent chromatin remodelling complex and are part of the RNA polymerase II holoenzyme complex. Sequence alignment of the CEN.PK113-7D *SWI1 *and *SNF11 *genes with the sequences from different sequenced *S. cerevisiae *strains showed a high diversity in gene and protein length (Figure 4 and Additional file 11: Figure S5).

Interestingly, the indels in *SWI1 *and *SNF11 *occur in tandem repeat regions. Such tandem repeats have been shown to predominantly occur in promoter regions and in genes encoding for transcription factors and cell-surface proteins [52]. In *S. cerevisiae *recombination of intragenic repeats of cell surface proteins has been suggested to be a reversible mechanism of evolutionary adaptation to the environment by creating cell surface diversity [57]. Although 128 genes that encode for transmembrane and cell wall proteins carried at least one indel, this number was not deemed significant (*p *< 0.05, Bonferroni corrected). However, similarly to analysis of human genome sequences [52], category enrichment analysis of genes with indels identified overrepresentation of the functional categories 'regulation of transcription' and 'DNA binding' (Figure 3). These repetitive sequences induce contraction and expansion of the gene length by recombination and slippage of the DNA polymerase [52]. In humans, repeat expansion in coding sequences has been implicated in several degenerative diseases as Huntington disease and spinocerebellar ataxias. The genetic cause of Huntington disease is a trinucleotide expansion in exon 1 of the *IT15 *gene that encodes for huntingtin, a protein involved in several cellular functions including transcription [58].

The severe effects of tandem repeat length variation in humans raises the question of the effect of this phenomenon in *S. cerevisiae*. Intriguingly, the physiological implications of these polymorphisms in transcription factors have not yet been studied. Dedicated research in the future should address this to understand the role of such variation.

### Absent and specific genes in CEN.PK113-7D relative to the reference S288C

A substantial number of *S. cerevisiae *genes are redundant as a result of genome duplication, thus allowing the loss of one copy of each duplicated gene. To systematically analyze the genes that were absent in CEN.PK113-7D sequence relative to S288C, a methodical search of S288C homologous genes with at least 95% identity in CEN.PK113-7D was performed. To prevent false negatives (i.e. genes called absent because they were not assembled), genes with a lower copy number (log_2 _ratio < -0.6) in CEN.PK113-7D than in S288C using the read mapping analysis were listed and compared to those S288C genes that did not present a homolog in CEN.PK113-7D. This analysis identified 83 genes absent in CEN.PK113-7D relative to S288C (Additional file 13: Table S6). Absence of 62 of these genes can be explained by the absence of several large fragments that, in strain S288C, are located in subtelomeric regions (Additional file 13: Table S6), such as on the left arm of CHRI (25 Kbp), on the left arm of CHRVIII (12 kb), on the left arm of CHRXII (17 kb) and on the right arm of CHRXIV (14 kb).

**Figure 5:**
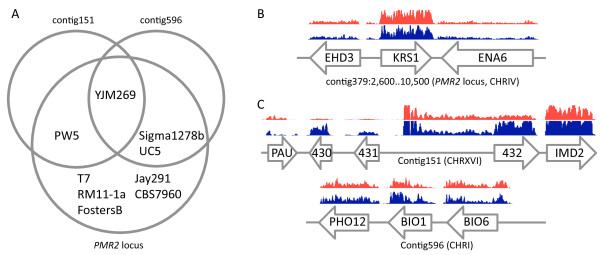
**Occurrence analysis of three regions present in the CEN.PK113-7D genome, but not in the S288C genome**. **A) **Venn diagram that represents the occurrence of the three regions over the available *S. cerevisiae *sequenced strains in Genbank (Additional file 12: Table S7). **B and C) **Annotation of the regions and RNA-seq expression profiles. RNA-seq data from glucose- and nitrogen limited anaerobic chemostat cultures (red and blue, respectively) were plotted (one bar every 10^th ^base) for the CEN.PK113-7D specific *ENA *locus (**B**) and the two specific contigs (**C**). Expression data, expressed as the number of times a base is covered by a read, are ranged from are [0-750] for contig379 and contig151 and [0-250] for contig596.

Only 21 deletions were found outside subtelomeric regions. Our assembly confirmed the size difference of the *PMR2 *locus in the CEN.PK113-7D strain [4]. In S288C the *PMR2 *locus on chromosome IV harbors five copies of *ENA *genes (*ENA1-ENA5*) that encode plasma membrane sodium pumping ATPases. Sequence analysis of the *PMR2 *locus in *S. cerevisiae *CEN.PK113-7D revealed the presence of a single and new *ENA6 *allele that showed substantial sequence differences, both at the nucleotide level and at the predicted amino acid sequence level, with previously described *ENA *genes (Figure 5). The presence of this single and atypical *ENA *gene correlated with hypersensitivity to sodium and, in particular, to lithium ions. This CEN.PK113-7D locus was also found in several industrial *S. cerevisiae *strains and natural isolates. The closest homolog was found in *S. cerevisiae *YJM269 (strain isolated from Blauer Portugieser grapes (GenBank: AEWN00000000.1)), which exhibited a complete coverage of the locus with an identity of 99% at the nucleotide level (Figure 5A).

The assembled CEN.PK113-7D genome sequence contained several sequences that are absent in S288C. The CEN.PK specific genes were organized in two large blocks: contig151 and contig596 (Figure 5C). The first block contig151, showed similarity with sequence data of *S. cerevisiae *strains YJM269 (79%) and PW5 (isolated from Nigerian Raphia palm wine) http://www.genome.wustl.edu/genomes/view/saccharomyces_cerevisiae_pw5 (63%) (Figure 5A). Of the five adjacent genes on contig151, the three new genes could not be functionally annotated. However, their expression could be confirmed by RNA-seq on samples taken from anaerobic carbon- and nitrogen-limited chemostats, commonly used cultivation conditions to study yeast physiology. Interestingly, uncharacterized genes 430, 431 and 432 on contig151 exhibited differential expression and were significantly up-regulated under nitrogen-limitation relative to carbon-limitation [59,60] (Figure 5C). Furthermore, Southern blotting of chromosomal separation gels established that contig151 could be placed on chromosome XVI (Additional file 14: Figure S3). These observations support the existence and functionality of these open reading frames in CEN.PK113-7D (Figure 5). Functional analysis of these new genes should be part of a larger, emerging challenge: the functional analysis of the growing pangenome of the species *S. cerevisiae *that arises from the advent of next-generation sequencing.

### CEN.PK113-7D specific sequences lead to biotin prototrophy

**Figure 6:**
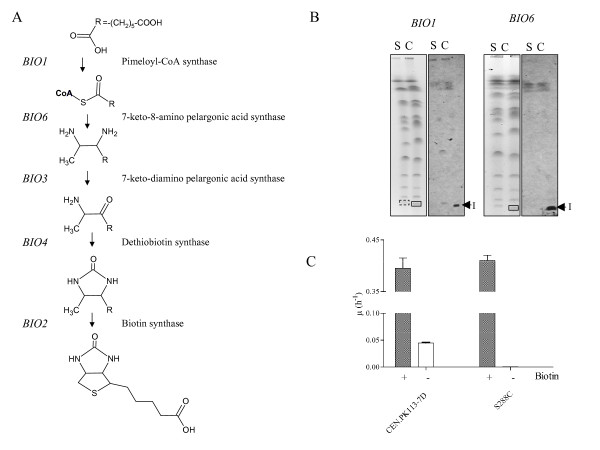
**Biotin biosynthesis characterization in CEN.PK113-7D and S288C**. **A) **Biotin biosynthesis pathway in CEN.PK113-7D. **B) **Southern blotting of S288C and CEN.PK113-7D chromosomes using specific probes for *BIO1 *and *BIO6 *genes. **C) **Maximal specific growth rate of CEN.PK113-7D and S288C in shake flask cultivations with synthetic medium [62] in absence (white bar), and presence (grey bar) of biotin. Growth rates were calculated from biomass measurements expressed as optical density measured at 660 nm.

The second sequence block present in CEN.PK113-7D but not in S288C is contig596 (Figure 5C). This contig contained three predicted open reading frames. The first of the three shared strong similarity (95% identity) with *PHO12 *that encodes an acid phosphatase. As mentioned earlier, *PHO12 *had three paralogs in the CEN.PK113-7D genome, which preclude the exact localization of this *PHO12 *family member found on contig596. The second and third open reading frames identified on this contig shared 72% identity with *BIO1 *(YJR154W) that encodes for a pimeloyl-CoA synthase and 95% identity with *BIO6 *that encodes for the following step in biotin biosynthesis, a 7-keto-8-amino pelargonic acid (KAPA) synthase (Figure 6A). Southern blotting of S288C and CEN.PK113-7D chromosomal separation gels indicated that these two open reading frames alongside the newly identified *PHO12 *family member were located on CHRI (Figure 6B).

The CEN.PK113-7D *BIO1 *and *BIO6 *did exhibit similarity within the *S. cerevisiae *pangenome. The genes are conserved (over 99%) in several other *S. cerevisiae *strains, such as Sigma1278b (laboratory strain used in pseudohyphal studies, GenBank: ACVY00000000.1), YJM269 and UC5 (isolated from Sene sake in Kurashi, Japan, GenBank: AFDD00000000.1) (Figure 5A). Although, the systematic sequence of S288C as reported in SGD indicates that these genes were absent, Hall and Dietrich (2007) identified two pairs of pseudo genes on CHRI ([YAR069W-A (*BIO6*) and YHR214W-F (*BIO8*)]) and CHRVIII [YAR070W-A (*BIO1*) and YHR214W-G (*BIO7*)] in S288C. Southern blot chromosomal localization of *BIO1 *and *BIO6 *genes in S288C and CEN.PK113-7D established that these two genes were indeed exclusively present in CEN.PK113-7D and were located on CHRI. If the pseudogene pairs were indeed present in S288C as previously proposed [61], they displayed very low degree of similarity. In addition *BIO1 *and *6 *transcripts were detected in glucose- and nitrogen-limited conditions (Figure 5C).

To verify whether these differences in genotype between S288C and CEN.PK113-7D were reflected in phenotype, we tested their growth in chemically defined medium with and without added biotin. After two consecutive shake-flask cultures in biotin-free medium to exhaust biotin reserves, while S288C strain failed to grow (μ < 0.0005 h^-1 ^in 100 hours), the CEN.PK113-7D strain successfully proliferated in the absence of biotin, at a specific growth rate of 0.045 h^-1 ^(compared to 0.38 h^-1 ^in presence of biotin). Biotin is an indispensable water-soluble vitamin that is mainly used as a cofactor by biotin-dependent carboxylases. In the literature, most *S. cerevisiae *strains are considered to have lost the ability to grow in absence of biotin. To complement this auxotrophy, biotin is routinely included in chemical defined fermentation media. Biotin needs to be dissolved at high pH at which it is not stable. This step is critical in medium preparation and can cause batch-to-batch variation. Strains prototrophic for biotin might offer a solution to this, and CEN.PK113-7D is an excellent model to evaluate the use of biotin prototrophic *S. cerevisiae *strains for industrial application. Such biotin prototrophy would be economically interesting. A quick estimate based on the amount of biotin used in chemically defined medium [62] designed to grow around 3 g.l^-1 ^biomass, an average catalog price for biotin of 100 €.g^-1 ^and a fermentation volume of 150 m^3 ^would reduce the medium cost by 1000€. This cost saving might be increased by 5 to 20-fold with media elaborated to grow higher biomass densities (e.g. fed-batch for protein production) [63].

### CEN.PK113-7D shares genomic material with both laboratory and industrial strains

**Figure 7:**
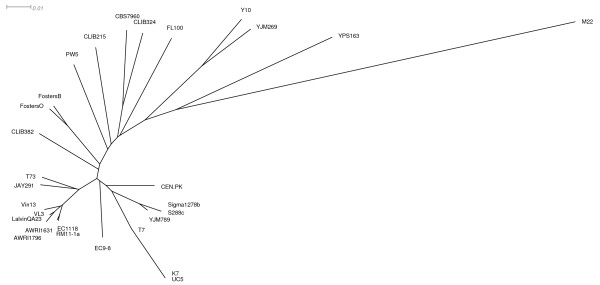
**Phylogenetic tree generated using UPGMA on genomic distances in the SplitsTree4 package **[65]**using the available *S. cerevisiae *genomes in Genbank (Additional file **12**: Table S7)**.

**Figure 8:**
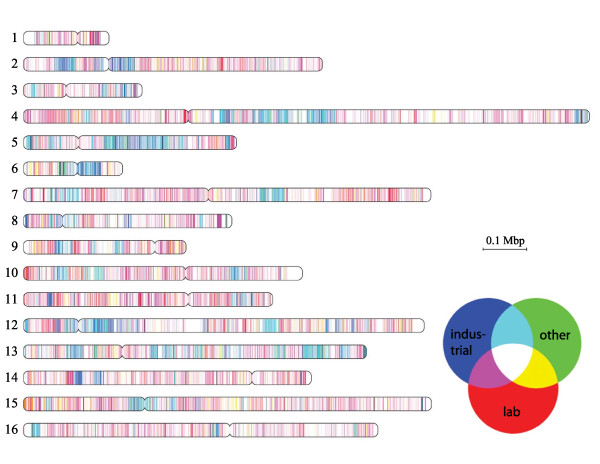
**Mosaic CEN.PK113-7D genome**. CEN.PK chromosomes colored by their identity to *S. cerevisiae *genomes, which were divided into the groups: laboratory (lab) (FL100:PRJNA60147, S288C:PRJNA128, Sigma1278b:PRJNA39317), industrial (e.g. wine, beer, bio-ethanol) (AWRI1631:PRJNA30553, AWRI796:PRJNA48559, CBS7960:PRJNA60391, CLIB382:PRJNA60145, EC1118:PRJEA37863, FostersB:PRJNA48569, FostersO:PRJNA48567, JAY291:PRJNA32809, Kyokai no. 7:PRJNA45827, Lalvin QA23:PRJNA48561, M22:PRJNA28815, PW5:PRJNA60181, RM11-1a:PRJNA13674, T73:PRJNA60195, UC5:PRJNA60197, Vin13:PRJNA48563, VL3:PRJNA48565, YJM269:PRJNA60389) and other (CLIB215:PRJNA60143, CLIB324:PRJNA60415, EC9-8:PRJNA73985, T7:PRJNA60387, Y10:PRJNA60201, YJM789:PRJNA13304, YPS163:PRJNA28813) (Additional file 12: Table S7). Each group was assigned one of the color channels of the RGB figure (red: lab, green: other and blue: industrial). The genome was divided in non-overlapping fragments of 1000 base pairs, represented by one pixel in the figure, which were aligned to the available *S. cerevisiae *genomes in GenBank (Additional file 12: Table S7). The identity of the best alignment in a group was set to be the value of the corresponding color channel. These values were scaled between 0 and 1; 0 meaning a maximal identity of 97% or lower, 1 meaning a maximal identity of 100%. For example, a white pixel color means 100% conservation of the fragment in all three groups. Blue and cyan mean conservation in industrial strains, but not in laboratory strains.

Our analysis so far focused on the genome comparison of CEN.PK113-7D and the main reference sequence of the laboratory strain S288C. Phylogenetic analysis that aimed at comparing CEN.PK113-7D genome to the other *S. cerevisiae *genomes available in GenBank (Additional file 12: Table S7), showed the CEN.PK113-7D genome was located in between the laboratory strains (S288C and Sigma1278b) and industrial strains, such as the bioethanol (JAY291), wine (EC1118, RM11-1a, AWRI1631, AWRI1796, Vin13, VL3, T73 and Lalvin QA23) and beer (CLIB382, FostersO and FostersB) strains (Figure 7). Pioneering works from Liti [2] and co-workers as weel as Schacherer and co workers [64] indeed performed similar analyses on a wide range of S. cerevisiae strains, however CEN.PK113-7D could never be positioned accurately. Liti and workers did not include CEN.PK in their work while Schacherer and co-workers did include it, but derived the phylogenetic distances between yeast strains from SNP distribution by array comparative genome hybridization experiments in which all strains were compared to the sequence of S288c excluding all sequences not found in S288c. Our approach based on full genome sequence of all involved strains depicts a clearer positioning of the CEN.PK strains in yeast phylogenetic tree. CEN.PK113-7D is not a typical laboratory strain, but has both features typically found in industrial *S. cerevisiae *strains and features that make it a suitable laboratory strain. Regions more closely related to either industrial strains or laboratory strains were distributed in a mosaic structure across the CEN.PK113-7D genome (Figure 8, Additional file 12: Table S7). The industrial background of CEN.PK113-7D may further be elucidated by sequencing its parental strains (ENY-WA-1A and MC996A), which have different phenotypes but are of unclear origin.

## Conclusion

We have sequenced, assembled and annotated the genome of *S. cerevisiae *CEN.PK113-7D. Complementary to previous re-sequencing efforts [35], our *de novo *assembly provides additional insight in still unexplored differences between *S. cerevisiae *strains, such as large indels. Comparisons of the *de novo *assembled genome of CEN.PK113-7D to other *S. cerevisiae *genomes revealed many unstable tandem repeats in genes involved in transcriptional regulation. Repeat expansion and contraction in genes coding for regulatory proteins may be a mechanism of evolving transcription regulation, reconfiguring binding affinity and specificity of transcription factors. From a metabolic engineering perspective, varying tandem repeat lengths may be a promising approach to 'tune' transcription regulation networks.

*De novo *assembly of the genome allowed us to uncover genes in CEN.PK113-7D that are absent in S288C and relate to phenotypic characteristics specific to CEN.PK113-7D such as maltose metabolism and biotin biosynthesis. These findings led to the surprising discovery that CEN.PK113-7D is biotin prototroph, a phenotypic trait potentially interesting for industrial application. Phylogenetic analysis based on whole-genome sequence established that CEN.PK113-7D is not solely related to laboratory strains like S288C, but also to industrial strains. These genetic specificities are the basis of the robust physiological performance of this strain, which is used as a yeast model for industrial applications. Together, these results contribute to the elucidation of the genetic diversity within the *S. cerevisiae *pangenome. In addition, a high-quality annotated genome of this popular model strain is now available as a resource to the yeast research community (GenBank BioProject PRJNA52955; http://cenpk.bt.tudelft.nl).

## Materials and methods

### DNA purification and sequencing

The genomic DNA of *S. cerevisiae *CEN.PK 113-7D [21] was purified as previously described [66]. High-throughput sequencing DNA libraries for Illumina (Illumina, San Diego, CA) and 454 (454 Life Sciences, Brandford, CT) sequencing were prepared according the manufacturer's recommendations. The Illumina paired-end library was sequenced on Genome Analyzer IIx (Illumina) with a read length of 50 bp at ServiceXS (Leiden, The Netherlands). The 454 library was sequenced on a GS FLX + system (454 Life Sciences) with an average read length of 350 bp at GATC-Biotech (Konstanz, Germany). The sequence reads were deposited in the Sequence Read Archive (SRA study accession: SRP011487.1).

### Assembly, scaffolding, annotation and RNAseq

The two sequencing methods used in this study yielded data with different properties in terms of error models and read length. Different assemblers, i.e. de Bruijn graph and overlap-layout-consensus assemblers, yield optimal results given these data types. We therefore chose a hybrid assembly strategy using the Velvet [67] assembler for the Illumina data and the Newbler assembler for the 454 data. The output of both assemblers was combined using the Maia algorithm [42]. Paired-end scaffolding was then performed using Velvet's scaffolder [67]. The resulting contigs were placed into chromosomal scaffolds based on homology with the S288C genome (*Saccharomyces *Genome Database, http://www.yeastgenome.org/, March 3^rd^, 2011) using MUMmer [68]. The chromosomal scaffolds were generated for visualization purposes. Variation analysis was performed on contigs rather than on scaffolds, as the bear no bias towards S288C.

ORF finding was performed using several tools combined in the Cyrille2 pipeline [69]. *Ab initio *gene prediction was performed using Augustus [70], SNAP [71] and GeneMark-S [72]. Comparative gene prediction was performed by mapping S288C ORFs, S288C proteins and UniProt fungal proteins with GenomeThreader [73].

The output of the above predictors was combined by Jigsaw [40] to predict the final ORFs for CEN.PK. These ORFs were aligned using BLAST [74] to the *Saccharomyces *Genome Database (SGD) to assign the name of the closest S288C homolog. The assembled data can be retrieved at GenBank [BioProject PRJNA52955] and at http://cenpk.bt.tudelft.nl).

#### RNAseq data

cDNAs from anaerobic carbon-limited, and anaerobic nitrogen-limited chemostat samples [60] were prepared as previously described [75]. The Illumina cDNA libraries were prepared according the manufacturer's recommendations. The libraries were sequenced on Genome Analyzer IIx with read length of 50 bp at Baseclear (Leiden, The Netherlands). The resulting reads were mapped onto the CEN.PK113-7D genome sequence using the Burrows-Wheeler Alignment tool BWA [45]. Visualization of the mapped reads was performed using the Integrated genomics viewer [76].

#### Gap and missing sequence analysis

We investigated the causes of fragmentation and potentially missing sequence between two CEN.PK113-7D contigs. Every CEN.PK113-7D contig was assigned a location in the S288C genome using alignment by MUMmer. Regions in S288C not covered by one of the CEN.PK113-7D contigs, i.e. the missing sequences or gaps, were analyzed. There are several possible causes for missing sequence: (1) the sequence is unique to S288C; (2) the regions were not well amplified in the PCR reaction in the sequencing instrument, because of too high or too low GC content; or (3) the reads are present but not assembled, possibly because of repeats. If the reads are present and the region is not repetitive, a contig break is unlikely. We therefore investigated the latter two causes of gaps: too low read coverage or repeats.

The Illumina reads were mapped to the S288C genome using BWA [45] and analyzed with Samtools [77]. First we counted the number of regions in the S288C genome between consecutively aligned CEN.PK113-7D contigs that contained at least one base not covered by any read. Such gaps we call *missing sequence*. Second, we determined if a repetitive sequence could be located in the gap. To this we aligned the S288C genome to itself using MUMmer. Regions were called *repetitive *if they had > 95% identity with sequence elsewhere in the genome. If a gap overlapped with one of these repetitive regions in S288C, it was labeled *unassembled*. The total size of *missing sequence *gaps and *unassembled *gaps was then tallied, providing insight in the causes of the fragmentation of the assembly.

### Variation analysis

#### SNV indels and novel DNA

CEN.PK113-7D contigs were aligned to S288C with MUMmer with an identity threshold of at least 97%. SNVs and indels were called from alignments with a non-ambiguous mapping. Small indels were detected as gapped alignments, maximized to a length of 90 bp by MUMmer's default settings.

We define a region in CEN.PK113-7D to be *unique *if it shows less than 95% identity to any region in S288C. To list these unique regions, first all non-unique regions were found by whole genome alignment of CEN.PK113-7D and S288C, keeping only alignments with more than 95% identity. For each position in the S288C genome only the best aligned CEN.PK113-7D region was considered; if other regions aligned, this was considered to be a duplication in CEN.PK113-7D, hence unique. All resulting unaligned regions were then called unique to CEN.PK113-7D and analyzed for genes. Conservation of these unique regions in other yeast strains was investigated by aligning them to the NCBI Whole-Genome-Shotgun Sequences database using blastn [74]. Species which contained a similar sequence were selected by sorting the hits on score and subsequently selecting the top hits with query coverage > 60% and identity > 90%.

Enrichment of Gene Ontology (GO) terms in the GO Fat subset [54] in the sets of SNVs and indels was performed in the DAVID functional annotation tool. *P*-values were calculated using the EASE score, a modified version of the Fisher exact test [78]. Bonferroni correction was applied by DAVID to account for multiple testing.

#### Deleted and duplicated genes

Copy number variation of regions in CEN.PK113-7D compared to S288C was investigated by mapping both S288C and CEN.PK113-7D reads to the S288C reference genome. The log_2_-ratio of the CEN.PK113-7D depth of coverage over that of S288C was calculated using CNV-seq version 0.2-6 [46] and normalized to an average of 1 per chromosome. For this analysis, 36 bp reads for both strains were obtained from [35]. The default CNV-seq threshold of 0.6 was used as a cut-off for the log_2_-ratio.

S288C genes that could not be mapped to the CEN.PK113-7D assembly by GenomeThreader with an identity score of at least 95% were labeled not present. An additional validation was performed to prevent false positive deletion calls of genes that should be present in the genome but are not assembled; using copy number variation between CEN.PK113-7D and S288C. Genes with a significantly lower copy number value in CEN.PK113-7D were considered not to be present in the data. The intersection of the set of genes not assembled and the set of genes not present in the data resulted in a high confidence list of deleted genes.

#### Transposon analysis

Ty retrotransposons mostly cause contig breaks in the assembly process, because their sequences are repetitive in the genome. The presence of the S288C transposons in the CEN.PK113-7D genome can be investigated by using these contig breaks. When inspecting the alignment of CEN.PK113-7D and S288C on locations these Ty elements in the S288C genome, three situations can be observed, the transposon is not present, therefore a gapped alignment of one contig spans the transposon location (GA) with a gap of size similar to the Ty retrotransposon size (~6-kbp); the transposon is present in CEN.PK113-7D, but unassembled, therefore two contigs align to both sides of the transposon location (CB); or the transposon is present in CEN.PK and assembled, therefore a gapless alignment is observed (AS). The first situation yields a not present call for the transposon, the second and third situation yield a present call. The presence of transposons unique to CEN.PK113-7D was not investigated, since this requires long insert mate-pair libraries. An estimate of the number of Ty elements in the CEN.PK113-7D genome for each member of the Ty family (Ty1-Ty5) was obtained by calculating the log_2_-ratio of the depth of coverage for each Ty element in S288C.

#### Southern blotting

The chromosomes of the CEN.PK113-7D strain were prepared and separated by clamped homogeneous electrical field (CHEF) electrophoresis as previously described in [38]. Transfer of the DNA from the gel to a nylon membrane (Amersham Hybond™-N+, GE Healthcare Europe GmbH, Diegem, Belgium), was performed as previously described in [79]. Genomic DNA probe fragments were amplified from genomic DNA of *S. cerevisiae *CEN.PK113-7D using Phusion Hot-Start Polymerase (Finnzymes, Landsmeer, The Netherlands) and the oligonucleotides listed in Additional file 5: Table S8. The probes were labelled according to the manufacturer's instructions (Amersham Gene Images AlkPhos Direct Labelling and Detection System, Buckinghamshire, UK). Hybridization was done overnight at 55°C in hybridization buffer (50% formamide, 5× Saline-Sodium Citrate (SSC) buffer, 2% blocking reagent (Roche), 0.1% Na-lauroylsarcosyl, 0.02% Sodium Dodecyl Sulfate (SDS)). Membranes were washed twice with primary wash buffer (2 M urea, 0.1% SDS, 50 mM sodium phosphate, 50 M sodium chloride, 1 mM magnesium chloride, 0.2% blocking reagent) for 15 minutes at 55°C and twice with secondary wash buffer (50 mM Tris base, 0.1 M sodium chloride, 2 mM magnesium chloride) for 5 minutes at room temperature. Digoxigenin-labelled probes were detected by chemiluminescence using CPD-star (Roche, Paris, France).

#### PCR amplification of the MAL loci

The genomic *MAL1, 2 *and *3 *loci were amplified from genomic DNA of *S. cerevisiae *CEN.PK113-7D using the Qiagen LongRange PCR kit (Qiagen, Hilden, Germany) according to manufacturer's instructions in a Biometra TGradient Thermocycler (Biometra, Gottingen, Germany). The following primer combinations were used for amplification: ZUO1 Fw/MALx2 Rv (*MAL1 *locus), YCR102W-A Fw/MALx2 Rv (*MAL2 *locus) and PHO89 Fw/MALx2 Rv (*MAL3 *locus) (Additional file 5: Table S8). Sequencing DNA libraries of each of *MAL *PCR locus for Illumina were prepared and the PCR products were sequenced on Genome Analyzer IIx with read length of 50 bp at Baseclear (Leiden, The Netherlands). The subsequent sequences were assembled using Newbler (454 Life Sciences, Brandford, CT).

#### Phylogenetic tree construction

The *S. cerevisiae *genomes were downloaded from GenBank (accession date: 21 Nov. 2011) (Additional file 12: Table S7). Similarities between all pairs of genomes were determined using MUMmer with the settings as recommended in [80]. Pairwise distances were calculated using the coverage distance function [81]. The phylogenetic tree was created by performing hierarchical clustering (UPGMA) with the SplitsTree4 package [65].

#### Strains and cultivation conditions

The prototrophic *S. cerevisiae *strains CEN.PK113-7D [21] and S288C (ATCC 204508) [82] were grown in liquid cultures. The shake-flask cultivations with biotin were performed in 500 ml flasks containing 100 ml of medium, which were incubated at 30°C on an orbital shaker set at 200-rpm. The composition of the synthetic medium (SM) was as follows: glucose (20 g.l^-1^), (NH_4_)_2_SO_4 _(5 g.l^-1^) KH_2_PO_4 _(3 g.l^-1^), MgSO_4 _(0.5 g.l^-1^), trace elements and vitamin solutions [62]. A separate biotin-free vitamin solution was used for growth in absence of biotin. The pH of the medium was adjusted to 5.0 and sterilized by autoclaving. Glucose was autoclaved separately. Vitamins were filter-sterilized and added to the medium. Growth of the various strains was monitored by OD measurements at 660 nm.

## Competing interests

The authors declare that they have no competing interests.

## Authors' contributions

JFN, DdR, JTP and JMD designed the experimental work. JFN, MvdB, MR and DdR carried-out the genome assembly. ED, RCvH, JFN, MvdB and DdR performed the genome annotation. SdK, LB, MAL, PDL performed molecular biology work. WV, JN, PK, WHMH, PKl, CJP, DP, PKö, JTP, JMD shared raw sequencing data. JFN, DdR, MR, JTP and JMD prepared the manuscript. All authors read and approved the final manuscript.

## Supplementary Material

Additional file 1 **Supplementary methods**. Click here for file

Additional file 2 **Table S1**. Repetitive transposon sequences were hard to assemble from whole genome shotgun data. Evidence of transposons was obtained in two ways. First, depth-of-coverage of CEN.PK113-7D and S288C reads on Ty retrotransposons sequences in the S288C genome was analysed. The number of retrotransposons was estimated from these ratios. Second, evidence for transposons in the assembly was obtained by counting the presence of contig breaks (CB) on transposon loci in S288C and the presence of assembled (AS) transposons (Figure S1). An assembled transposon locus with a gapped alignment (GA) around the transposon sequence in S288C indicates the transposon is absent from the CEN.PK genome. Click here for file

Additional file 3 **Figure S1**. Analysis of transposon composition by alignment of the CEN.PK113-7D and S288c genomes. When an S288c transposon is not present in CEN.PK113-7D it results in a gapped alignment (GA) of about 6 Kbp. Transposons that are present can cause contig breaks (CB) in the assembly. Only YCLWTy5-1 was fully assembled (AS). Click here for file

Additional file 4 **Figure S2**. Chromosome separation gel with *RDL1 *and *PHO12 *probed. Click here for file

Additional file 5 **Table S8**. Primer used in this study. Click here for file

Additional file 6 **Table S2**. SNVs in genes in CEN.PK113-7D compared to S288C. Click here for file

Additional file 7 **Table S4**. Mutations in the galactose uptake and ergosterol biosynthesis pathways compared to the SNVs found previously in CEN.PK113-7D [35]. Click here for file

Additional file 8 **Table S5**. Mutations found in genes in the cAMP signaling pathway. Click here for file

Additional file 9 **Figure S4**. Differences between CEN.PK113-7D and S288C in the MAPK signaling pathway. Click here for file

Additional file 10 **Table S3**. Indels in genes in CEN.PK113-7D compared to S288C. Click here for file

Additional file 11 **Figure S5**. Multiple sequence alignment of Snf11p and Swi1p. Click here for file

Additional file 12 **Table S7**. *S. cerevisiae *with an assembled genome deposited in GenBank. The classification assigned in the 'group' column was used to generate Figure 8. Click here for file

Additional file 13 **Table S6**. List of deleted genes, which is defined as not having a homologous hit in the CEN.PK113-7D genome for at least 95% and having a CEN.PK113-7D/S288c log2 ratio of less then -0.6. The *PMR2 *locus has a blue background color. Click here for file

Additional file 14 **Figure S3**. Chromosome separation gel with contig151 probed. Click here for file
