# Supplementary material for: De novo sequencing, assembly and analysis of the genome of the laboratory strain Saccharomyces cerevisiae CEN.PK113-7D, a model for modern industrial biotechnology
Source: Microb Cell Fact. 2012 Mar 26;11:36. doi: 10.1186/1475-2859-11-36 (PMC3364882; doi:10.1186/1475-2859-11-36)
Supplement: Additional file 1 — Supplementary methods. [file 1475-2859-11-36-S1.DOCX]

# Supplementary Materials & Methods

The two sequencing data types have different properties in terms of error models and read length. Different assemblers yield optimal results given these data types. A hybrid assembly strategy merging the output of multiple assemblers was therefore chosen. For the 454 data an overlap-layout-consensus assembler was used (Newbler in its default mode). For the Illumina data a *de Bruijn* graph assembler was used (Velvet). Velvet was run in two modes. First, only the Illumina data was used in the input; second, the 454 contigs we included as ‘long reads’. The Velvet parameters were optimized using the velvetOptimizer perl script. Paired-end scaffolding was applied in both Velvet assemblies.

All assemblies were tiled on the *S. cerevisiae* S288c reference genome using MUMmer. The contigs were aligned to the reference using ‘nucmer’ and subsequently filtered with ‘delta-filter -1’ to give a 1-to-1 alignment allowing for rearrangements. The show-tiling command generated the tiling using default settings, except that minimum contig coverage of 80% was required.

The Newbler contigs gave the best tiling result in terms of genome coverage. This tiling together with overlaps between contigs in the different assemblies were used as input for MAIA to combine the assemblies. A minimum overlap of 150 bp between the contigs was required. The maximum non-aligned overlap was set to 30.

The assembly combination with MAIA resulted in 468 paired-end scaffolds with a total length of 11.2 Mbp. The Newbler and Velvet contigs larger than 200 bp that were not yet contained in the MAIA assembler were manually added to the assembly. In order to do determine non-contained contigs the Newbler contigs were aligned to the MAIA assembly using nucmer, non-contained contigs were added and subsequently this procedure was repeated for the Velvet contigs. The resulting 565 paired-end scaffolds with a total length of 11.6 Mbp were place into sixteen chromosomal and one mitochondrial scaffold. The remaining 55 contigs containing 55 Kbp were placed into a scaffold named ‘chromosome 0’, separated by 200 bp. This was done for visualization purposes in GBrowse.

The 454 data used in the assembly is known to be sensitive to homopolymer errors. These errors were corrected using the k-mer correction tool (Datema et al.). After correction the assembly was annotated using Cyrille2 pipeline. Genes in the CEN.PK genome were located using a combination of tools. Both *ab initio* and comparative gene predictors were applied. The predicted gene models were combined using Jigsaw [3]. The resulting annotated genome will be made available through Gbrowse [4] (Fig. 2).

# Supplementary tables and figures

**Table S1** Repetitive transposon sequences were hard to assemble from whole genome shotgun data. Evidence of transposons was obtained in two ways. First, depth-of-coverage of CEN.PK and S288C reads on Ty retrotransposons sequences in the S288C genome was analysed. Log_2_-ratio's were calculated using CNV-seq ([Xie & Tammi, 2009](#_ENREF_3)). The number of retrotransposons was estimated from these ratios. Second, evidence for transposons in the assembly was obtained by counting the presence of contig breaks (CB) on transposon loci in S288C and the presence of assembled (AS) transposons (Figure S1). An assembled transposon locus with a gapped alignment (GA) around the transposon sequence in S288C indicates the transposon is absent from the CEN.PK genome.

**Table S2** (Excel file) SNVs in genes in CEN.PK compared to S288c found by aligning the CEN.PK assembled genome to the S288c reference genome with MUMmer ([Kurtz*, et al.*, 2004](#_ENREF_2)).

**Table S3** (Excel file) Indels in genes in CEN.PK compared to S288c found by aligning the CEN.PK assembled genome to the S288c reference genome with MUMmer ([Delcher*, et al.*, 2002](#_ENREF_1)) ([Kurtz*, et al.*, 2004](#_ENREF_2)).

**Table S4** (Excel file) Mutations in the galactose uptake and ergosterol biosynthesis pathways compared to the SNVs found previously in CEN.PK Otero et al (2010).

**Table S5** Mutations found in genes in the cAMP signaling pathway. The genes that were considered to be part of the cAMP signaling pathway are listed in Figure 2.

**Table S6** (Excel file) List of deleted genes, which is defined as not having a homologous hit in the CEN.PK genome for at least 95% and having a CEN.PK/S288c log2 ratio of less then -0.6. The *PMR2* locus has a blue background color.

**Table S7** *S. cerevisiae* with an assembled genome deposited in GenBank. The classification assigned in the ‘group’ column was used to generate Figure 8.

**Table S8** Primer used in this study.

**Figure S1** Analysis of transposon composition by alignment of the CEN.PK and S288c genomes. When an S288c transposon is not present in CEN.PK it results in a gapped alignment (GA) of about 6 Kbp. Transposons that are present can cause contig breaks (CB) in the assembly. Only YCLWTy5-1 was fully assembled (AS).

**Figure S2** Chromosome separation gel with *RDL1* and *PHO12* probed.

**Figure S3** Chromosome separation gel with Contig00483 probed.

**Figure S4** Differences between CEN.PK and S288c in the MAPK signaling pathway.

**References supplemental material**

Delcher AL, Phillippy A, Carlton J & Salzberg SL (2002) Fast algorithms for large-scale genome alignment and comparison. *Nucleic acids research* **30**: 2478-2483.

Kurtz S, Phillippy A, Delcher AL, Smoot M, Shumway M, Antonescu C & Salzberg SL (2004) Versatile and open software for comparing large genomes. *Genome biology* **5**: R12.

Xie C & Tammi MT (2009) CNV-seq, a new method to detect copy number variation using high-throughput sequencing. *BMC bioinformatics* **10**: 80.
