# Supplementary material for: De novo sequencing, assembly and analysis of the genome of the laboratory strain Saccharomyces cerevisiae CEN.PK113-7D, a model for modern industrial biotechnology
Source: Microb Cell Fact. 2012 Mar 26;11:36. doi: 10.1186/1475-2859-11-36 (PMC3364882; doi:10.1186/1475-2859-11-36)
Supplement: Additional file 2 — Table S1. Repetitive transposon sequences were hard to assemble from whole genome shotgun data. Evidence of transposons was obtained in two ways. First, depth-of-coverage of CEN.PK113-7D and S288C reads on Ty retrotransposons sequences in the S288C genome was analysed. The number of retrotransposons was estimated from these ratios. Second, evidence for transposons in the assembly was obtained by counting the presence of contig breaks (CB) on transposon loci in S288C and the presence of assembled (AS) transposons (Figure S1). An assembled transposon locus with a gapped alignment (GA) around the transposon sequence in S288C indicates the transposon is absent from the CEN.PK genome. [file 1475-2859-11-36-S2.DOC]

**Table S1:**

| Type | log2 | # in S288c | # in CEN.PK | Not present, gapped alignment (GA) | Possibly present, contig break (CB) | Present, assembled (AS) |
| --- | --- | --- | --- | --- | --- | --- |
| Ty1 | -0.4 | 31 | 23 | 9 | 22 | 0 |
| Ty2 | -0.1 | 13 | 12 | 1 | 12 | 0 |
| Ty3 | 0 | 2 | 2 | 0 | 2 | 0 |
| Ty4 | -1.5 | 3 | 1 | 0 | 3 | 0 |
| Ty5 | 0 | 1 | 1 | 0 | 0 | 1 |
| Total | -0.4 | 50 | 38 | 10 | 39 | 1 |
