# Supplementary figures and images for: De novo sequencing, assembly and analysis of the genome of the laboratory strain Saccharomyces cerevisiae CEN.PK113-7D, a model for modern industrial biotechnology
Source: Microb Cell Fact. 2012 Mar 26;11:36. doi: 10.1186/1475-2859-11-36 (PMC3364882; doi:10.1186/1475-2859-11-36)

GA

CB

AS

CEN.PK

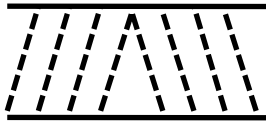

S288c

Ty

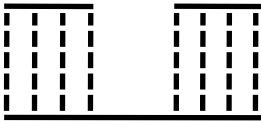

Ty

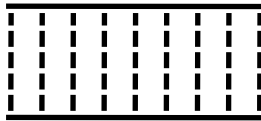

Ty

Supplement: Additional file 3 — Figure S1. Analysis of transposon composition by alignment of the CEN.PK113-7D and S288c genomes. When an S288c transposon is not present in CEN.PK113-7D it results in a gapped alignment (GA) of about 6 Kbp. Transposons that are present can cause contig breaks (CB) in the assembly. Only YCLWTy5-1 was fully assembled (AS). [file 1475-2859-11-36-S3.PDF]

*PHO12*

*RDL1*

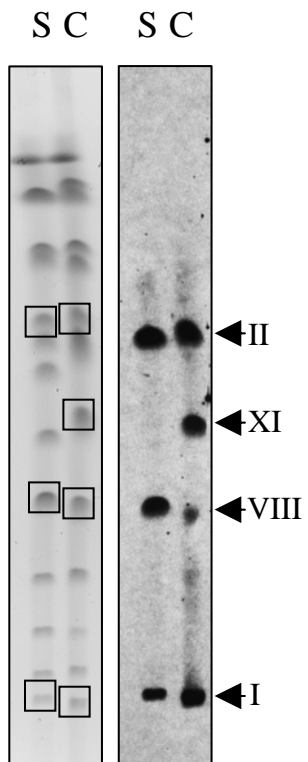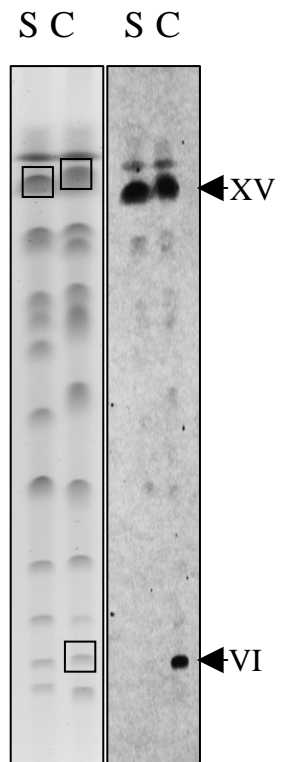

Supplement: Additional file 4 — Figure S2. Chromosome separation gel with RDL1 and PHO12 probed. [file 1475-2859-11-36-S4.PDF]

# MAPK SIGNALING PATHWAY

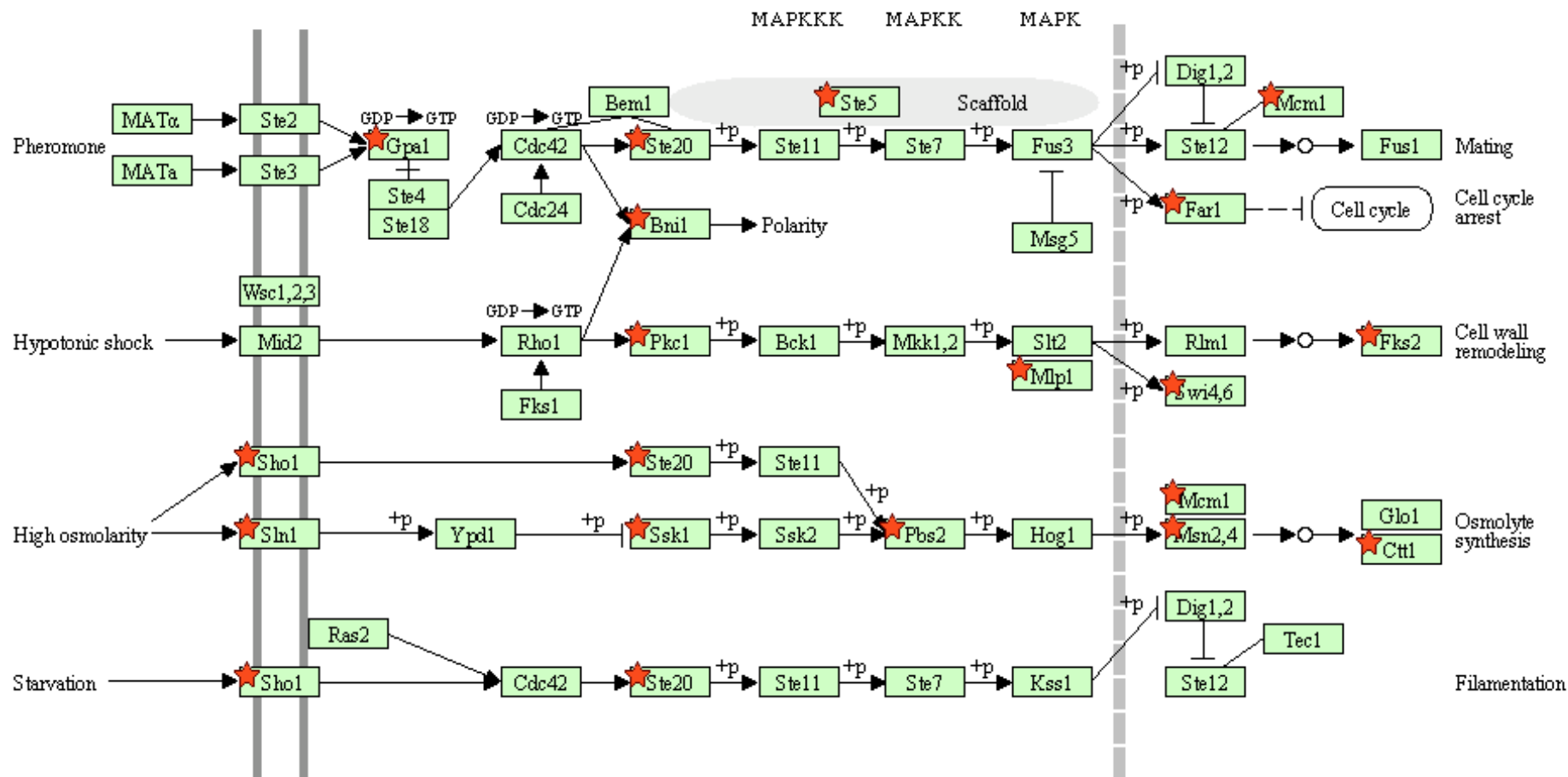

Supplement: Additional file 9 — Figure S4. Differences between CEN.PK113-7D and S288C in the MAPK signaling pathway. [file 1475-2859-11-36-S9.PDF]

S C

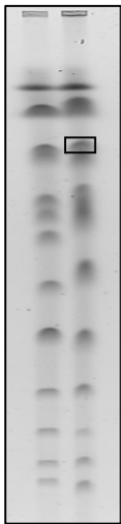

S C

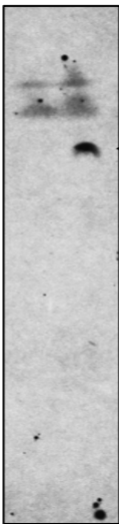

← XVI

Supplement: Additional file 14 — Figure S3. Chromosome separation gel with contig151 probed. [file 1475-2859-11-36-S14.PDF]
