# Supplementary material for: De novo sequencing, assembly and analysis of the genome of the laboratory strain Saccharomyces cerevisiae CEN.PK113-7D, a model for modern industrial biotechnology
Source: Microb Cell Fact. 2012 Mar 26;11:36. doi: 10.1186/1475-2859-11-36 (PMC3364882; doi:10.1186/1475-2859-11-36)
Supplement: Additional file 5 — Table S8. Primer used in this study. [file 1475-2859-11-36-S5.DOC]

**Table S8** Primer used in this study.

Primers used to amplify genomic DNA probe fragments from genomic DNA of *S.cerevisiae* CEN.PK113-7D using Phusion Hot-Start Polymerase (Finnzymes, Landsmeer, The Netherlands).

| **Probe** | **Forward primer (from 5' to 3')** | **Reverse primer (from 5' to 3')** |
| --- | --- | --- |
| *MAL32* | GCAGAAGGGCAATCTTTGAAAGTG | AGCAGCAAACAGCGTCTTGTC |
| *BIO1* | GCTAGGGTTCGCAATATGTCCTGG | CCACCACCTCATAAAGTTTACTGG |
| *BIO2* | CAACCATACCTAAAGTAACACACG | CCTCAGAAGAGCTAACTTAGCCG |
| *BIO6* | GAAAGGTCTAATCCACGCACCTGCC | GCTGGTGGTGCGTTCAGCAAGG |
| *IMA1* | GTTCTGGAAGAGGCATCTAC | CTCCGACGAAACTAAGAGAC |
| *PHO12* | CTGACGCTGCTGACGTTACAGACC | CGCTTCTTTGGTTAATGCAGGTACCATACC |
| *RDL1* | TCCATAAGGCCCAAATCC | CACAGCCAATGCAATAGG |
| Contig00483 | GGATATTCCTTGCCAGAGCACC | TGGATTGGCATGGTATATCC |

Primers used to amplify the MAL loci in CEN.PK and S288c.

| Primer | Sequence (5`🡪3`) |
| --- | --- |
| YCR102W-A Fw | GAGCTCTAACGATGAACCGTTACC |
| PHO89 Fw | CACACCAAAGCGAGGAGCATTC |
| ZUO1 Fw | TCTGGCACCGGCTTCTCTTTC |
| MALx2 Rv | AAGCAGCAAACAGCGTCTTGTC |

Primers used to amplify the IRA2 gene to validate the indel in CEN.PK sequence relative to the S288c sequence.

| Primer | Sequence (5`🡪3`) |
| --- | --- |
| IRA2 SNP FW | GATTTACGCCGGTATCGTTG |
| IRA2 SNP RV | GGCTGTGAGTTCTGGATTAG |
