# Supplementary material for: De novo sequencing, assembly and analysis of the genome of the laboratory strain Saccharomyces cerevisiae CEN.PK113-7D, a model for modern industrial biotechnology
Source: Microb Cell Fact. 2012 Mar 26;11:36. doi: 10.1186/1475-2859-11-36 (PMC3364882; doi:10.1186/1475-2859-11-36)
Supplement: Additional file 8 — Table S5. Mutations found in genes in the cAMP signaling pathway. [file 1475-2859-11-36-S8.DOC]

**Table S5** Mutations found in genes in the cAMP signaling pathway. The genes that were considered to be part of the cAMP signaling pathway are listed in Figure 2.

| **Gene** | **Mutation count** | **Mutations** |
| --- | --- | --- |
| Plc1 | 34x Sense | L35I, D36G, F37F, D50D, K52K, A72A, N93D, L102L, R114R, S143S, K144K, L146L, T159M, Y163Y, D175D, A181T, F232F, L280S, D282D, L289L, V339V, Y347H, G360G, K369K, E379E, E411E, C438C, P464P, E493E, G528D, A581V, L615L, R618H, V666V, L704L, K707K, S730N, R738R, I739I, L748L, N749N, V764V, M774T, I778I, K780K, Q829P, Q829Q |
|  | 13x Missense |  |
|  |  |  |
| Gpa2 | 13x Sense | P161P, Q193Q, M200V, S221S, G235G, P236P, L270L, E341E, L343L, D368D, A395A, A396A, L406L, R408R, T441R |
|  | 2x Missense |  |
| Gpb2 | 9x Sense | D217N, L327L, T374T, M474T, K534K, G536G, S552R, A674T, V741V, P777S, D826D, T827T, V832I, L848L, S875S |
|  | 6x Missense |  |
| Cyr1 | 1x Missense | K1876M |
| Ira2 | 1x Indel | .7014A |
| Flo8 | 1x indel (24 bp and no frame-shift) | GCTGCTGTTGCTGCTGCTGCTGTT2243. |
| Ste20 | 8x Missense, 13xSense |  |
