# Supplementary material for: De novo sequencing, assembly and analysis of the genome of the laboratory strain Saccharomyces cerevisiae CEN.PK113-7D, a model for modern industrial biotechnology
Source: Microb Cell Fact. 2012 Mar 26;11:36. doi: 10.1186/1475-2859-11-36 (PMC3364882; doi:10.1186/1475-2859-11-36)
Supplement: Additional file 11 — Figure S5. Multiple sequence alignment of Snf11p and Swi1p. [file 1475-2859-11-36-S11.PDF]

Snf11p

YJM789	SNTNTNTENENRNTGAGVDVNT**NANATANATA**------------ELNLPTVDEQRQYKVQLLLHINSILLARV
Kyokai no.7	SNTNTNTENENRNTGAGVDVNT**NANANANATANATA**--------ELNLPTVDEQRQYKIQLLLHINSILLARV
FostersO	SNTNTNTENENXNTGAGVDVNT**NANAXANATANATA**--------ELNLPTVDEQRQYKVQLLLHINSILLARV
FostersB	SNTNTNTENENRNTGAGVDVNT**NANANANATANATA**--------ELNLPTVDEQRQYKVQLLLHINSILLARV
CEN.PK	SNTNTNTENENRNTGAGVDVNT**NANANANATANATANATA**----ELNLPTVDEQRQYKVQLLLHINSILLARV
S288c	SNTNTNTENENRNTGAGVDVNT**NANANANATANATANATANATA**ELNLPTVDEQRQYKVQLLLHINSILLARV
EC1118	SNTNTNTENENRNTGAGVDVNT**NANATANATANATANATANATA**ELNLPTVDEQRQYKVQLLLHINSILLARV

Swi1p

YJM789	QQKQQQRFQAL**QQQQQQQQQQQQQQQQQ--QQQQQQQQNQQPQQQNQQPQQQQQQQQQQQQQQQ**NPKFLQSQR
Kyokai no.7	QQKQQQRFQAL**QQQQQQQQQ-----------NQQPQQQNQQPQQQ-----QQQQQQQQQQQQQQ**NPKFLQSQR
CEN.PK	QQKQQQRFQAL**QQQQQQQQQQQQQQQQQQQQNQQPQQQNQQPQQQ-----QQQQQQQQQQQQQQ**NPKFLQSQR
S288c	QQKQQQRFQAL**QQQQQQQQ------------NQQQ----------------QNQQPQQQQQQQQ**NPKFLQSQR
EC1118	QQKQQQRFQAL**QQQQQQQQQ-----------QQQQQQQQQQNQQQ------QNQQPQQQQQQQQ**NPKFLQSQR
JAY291	QQKQQQRFQAL**QQQQQQQQQ-----------QQQQQQQQQQNQQQ------QNQQPQQQQQQQQ**NPKFLQSQR
RM11-1a	QQKQQQRFQAL**QQQQQQQQQ-----------QQQQQQQQQQQQQQ------QNQQPQQQQQQQQ**NPKFLQSQR
