# Supplementary material for: De novo sequencing, assembly and analysis of the genome of the laboratory strain Saccharomyces cerevisiae CEN.PK113-7D, a model for modern industrial biotechnology
Source: Microb Cell Fact. 2012 Mar 26;11:36. doi: 10.1186/1475-2859-11-36 (PMC3364882; doi:10.1186/1475-2859-11-36)
Supplement: Additional file 12 — Table S7. S. cerevisiae with an assembled genome deposited in GenBank. The classification assigned in the 'group' column was used to generate Figure 8. [file 1475-2859-11-36-S12.DOC]

**Table S7** *S. cerevisiae* with an assembled genome deposited in GenBank. The classification assigned in the ‘group’ column was used to generate Figure 8.

| **Tag** | **BioProject** | **Publication** | **Group** |
| --- | --- | --- | --- |
| FL100 | PRJNA60147 | Saccharomyces cerevisiae strain project | Laboratory |
| S288C | PRJNA128 | Goffeau et al, 1996 | Laboratory |
| Sigma1278b | PRJNA39317 | Dowell et al, 2010 | Laboratory |
| CLIB215 | PRJNA60143 | Saccharomyces cerevisiae strain project | Other |
| CLIB324 | PRJNA60415 | Saccharomyces cerevisiae strain project | Other |
| EC9-8 | PRJNA73985 | Submitted, 2011 | Other |
| T7 | PRJNA60387 | Saccharomyces cerevisiae strain project | Other |
| Y10 | PRJNA60201 | Saccharomyces cerevisiae strain project | Other |
| YJM789 | PRJNA13304 | Wei et al, 2007 | Other |
| YPS163 | PRJNA28813 | Doniger et al, 2008 | Other |
| AWRI1631 | PRJNA30553 | Borneman et al, 2008 | Wine/Beer/Industrial |
| AWRI796 | PRJNA48559 | Borneman et al, 2011 | Wine/Beer/Industrial |
| CBS 7960 | PRJNA60391 | Saccharomyces cerevisiae strain project | Wine/Beer/Industrial |
| CLIB382 | PRJNA60145 | Saccharomyces cerevisiae strain project | Wine/Beer/Industrial |
| EC1118 | PRJEA37863 | Novo et al, 2009 | Wine/Beer/Industrial |
| FostersB | PRJNA48569 | Borneman et al, 2011 | Wine/Beer/Industrial |
| FostersO | PRJNA48567 | Borneman et al, 2011 | Wine/Beer/Industrial |
| JAY291 | PRJNA32809 | Argueso et al, 2009 | Wine/Beer/Industrial |
| Kyokai no. 7 | PRJNA45827 | Akao et al, 2011 | Wine/Beer/Industrial |
| Lalvin QA23 | PRJNA48561 | Borneman et al, 2011 | Wine/Beer/Industrial |
| M22 | PRJNA28815 | Doniger et al, 2008 | Wine/Beer/Industrial |
| PW5 | PRJNA60181 | Saccharomyces cerevisiae strain project | Wine/Beer/Industrial |
| RM11-1a | PRJNA13674 | *Saccharomyces cerevisiae* RM11-1a Sequencing Project. Broad Institute of Harvard and MIT | Wine/Beer/Industrial |
| T73 | PRJNA60195 | Saccharomyces cerevisiae strain project | Wine/Beer/Industrial |
| UC5 | PRJNA60197 | Saccharomyces cerevisiae strain project | Wine/Beer/Industrial |
| Vin13 | PRJNA48563 | Borneman et al, 2011 | Wine/Beer/Industrial |
| VL3 | PRJNA48565 | Borneman et al, 2011 | Wine/Beer/Industrial |
| YJM269 | PRJNA60389 | Saccharomyces cerevisiae strain project | Wine/Beer/Industrial |
